# NK Cell Disfunction in Atopic Dermatitis: A Missing Link Between Type 2 Inflammation, Microbial Dysbiosis and Antiviral Immunity

**DOI:** 10.3390/ijms27146477

**Published:** 2026-07-21

**Authors:** Maja Jakoniuk, Katarzyna Kler, Anna Kler, Kacper Rak, Małgorzata Ponikowska

**Affiliations:** 1 Faculty of Medicine, Wroclaw Medical University, Wybrzeze L. Pasteura 1, 50-367 Wroclaw, Poland; 2 University Centre of General Dermatology and Oncodermatology, Wroclaw Medical University, 50-556 Wroclaw, Poland

**Keywords:** atopic dermatitis, natural killer cells, innate immunity, skin microbiome, *Staphylococcus aureus*, targeted therapy, dupilumab, IL-15 superagonist, viral complications

## Abstract

Atopic dermatitis (AD) is a prevalent chronic inflammatory skin disorder driven by epidermal barrier defects and dysregulated Th2 cell responses. While therapies primarily address adaptive immunity, the role of Natural Killer (NK) cells remains underappreciated. This review analyzes NK cell dysfunctions in AD pathogenesis, evaluating their contributions to compromised skin immunity, microbial dysbiosis, and secondary infections. Accumulating evidence reveals a systemic deficiency of mature, cytotoxic CD56^dim^ and NKp80+ NK cell subsets in peripheral blood, correlating with disease severity. Within the cutaneous microenvironment, *Staphylococcus aureus* subverts defenses by utilizing leukocidins to lyse mature NK cells, while superantigens drive an aberrant, pro-inflammatory CD57^−^NKG2+ phenotype, exacerbating inflammation. Furthermore, localized exhaustion of functional NK cells and failure to produce interferon-gamma directly explains AD patients’ unique susceptibility to severe viral complications like eczema herpeticum. Importantly, treatments such as dupilumab and gut microbiota transplantations demonstrate that these NK cell aberrations are reversible, shifting immunity toward a normalized regulatory state. In conclusion, the NK cell compartment represents a vital regulatory axis bridging innate and adaptive immunity. Targeting this axis, particularly through IL-15 superagonists, offers a promising therapeutic frontier to suppress type 2 inflammation and restore antimicrobial defenses.

## 1. Introduction

Atopic dermatitis (AD) is one of the most common chronic inflammatory skin diseases, affecting approximately 13% of children and 5% of adults worldwide [1]. The disease typically develops during early childhood but may persist into adulthood or arise de novo later in life [2]. Clinically, AD is characterized by recurrent eczematous lesions, intense pruritus, epidermal barrier dysfunction, and a substantial impact on quality of life [3,4,5]. Beyond its cutaneous manifestations, AD is associated with increased susceptibility to allergic diseases, recurrent infections, and systemic immune abnormalities [4,5].

The pathogenesis of AD is complex and multifactorial, involving a dynamic interplay between epidermal barrier impairment, genetic predisposition, microbial dysbiosis, and immune dysregulation [6]. Traditionally, AD has been viewed as a predominantly type 2-driven inflammatory disorder, characterized by excessive activation of Th2 cells, type 2 innate lymphoid cells (ILC2s), and the overproduction of cytokines such as IL-4, IL-13, and IL-31 [6,7]. This historical view has since evolved into an integrated model that recognizes innate immunity as a critical orchestrator connecting the epithelial barrier with the adaptive immune axis [6]. These pathways contribute to chronic inflammation, pruritus, and progressive disruption of barrier integrity. However, the persistent susceptibility of patients with AD to microbial colonization and viral infections suggests that additional mechanisms beyond adaptive type 2 immunity contribute to disease pathogenesis.

Increasing evidence indicates that abnormalities within the innate immune compartment may represent a critical but underappreciated component of AD. Among innate immune cells, Natural Killer (NK) cells have emerged as potential regulators of the skin barrier–microbiome–immune axis. NK cells are cytotoxic innate lymphocytes capable of eliminating virally infected and transformed cells without prior antigen sensitization while simultaneously shaping adaptive immune responses through cytokine production, particularly interferon-gamma (IFN-γ) [8,9,10,11]. Their activity is tightly controlled by a balance of activating and inhibitory receptors, allowing rapid responses to cellular stress while maintaining self-tolerance [8,10,11].

In addition to their well-established antiviral functions, NK cells contribute to tissue homeostasis, immune regulation, and host defense at barrier surfaces, including the skin [9,11]. Distinct populations of tissue-resident NK cells have been identified within the dermis, where they participate in the regulation of local inflammatory responses and interactions with the cutaneous microbiota [9]. Emerging studies demonstrate that AD is associated with profound alterations in NK-cell biology, including depletion of mature cytotoxic subsets, impaired IFN-γ production, expansion of dysfunctional NK-cell populations, and disruption of regulatory NK-cell pathways [12,13,14]. Importantly, these abnormalities correlate with disease severity, microbial dysbiosis, epidermal barrier dysfunction, and increased susceptibility to severe viral infections such as eczema herpeticum [12,15].

Notably, NK-cell dysfunction appears to occupy a strategic position at the intersection of several major pathogenic hallmarks of AD. Quantitative and functional defects of NK cells may simultaneously facilitate type 2 inflammation, promote *S. aureus* colonization, impair antiviral immunity, and disrupt regulatory interactions with ILC2s and other innate immune populations. These observations support the concept that NK-cell dysfunction may represent a previously underrecognized mechanistic link connecting chronic inflammation, microbial dysbiosis, and infectious susceptibility in AD.

The therapeutic landscape of AD has undergone a profound transformation with the introduction of targeted biologic therapies and Janus kinase inhibitors. While the clinical success of agents targeting the IL-4/IL-13 axis has confirmed the central role of type 2 inflammation, emerging evidence suggests that successful treatment is also accompanied by partial restoration of NK-cell homeostasis [12,16]. These findings raise the possibility that NK cells may represent not only biomarkers of disease activity, but also future therapeutic targets.

This review establishes a new conceptual framework positioning NK-cell dysfunction as the critical missing link that bridges the major pillars of AD pathogenesis: type 2 inflammation, epidermal barrier collapse, and infectious susceptibility. By evaluating their interactions with *S. aureus* and the cutaneous microenvironment, we highlight how restoring NK-cell homeostasis represents a promising, underappreciated frontier in targeted AD therapeutics.

## 2. Materials and Methods

This review was conducted as a narrative overview of the quantitative and functional dysfunctions of Natural Killer (NK) cells in the pathogenesis of atopic dermatitis (AD), their impact on skin immunity, microbial dysbiosis, and their potential as novel therapeutic targets. Relevant articles were identified by performing a structured search of the PubMed, Google Scholar, and Science.org databases. To ensure the highest quality and relevance of the data, the search prioritized the latest original clinical and basic science research, as well as comprehensive review articles published up to May 2026. The following keywords and their combinations were used: “atopic dermatitis,” “Natural Killer cells,” “NK cells,” “innate immunity,” “skin microbiome,” “*S. aureus*,” “targeted therapy,” “dupilumab,” “IL-15 superagonist,” and “viral complications.”

Inclusion criteria were as follows:

- Peer-reviewed articles in English;
- The latest original clinical trials, in vivo/in vitro experimental studies, and systematic reviews;
- Studies providing significant insights into the mechanisms of NK cell depletion, their interactions with the cutaneous microenvironment (including *S. aureus*), and treatment algorithms modulating the NK-ILC axis in AD.

Exclusion criteria included:

- Non-peer-reviewed articles;
- Case reports or conference abstracts without full data;
- Purely computational studies without biological validation.

Reference lists of the included articles were also manually screened to identify additional relevant publications. Although the main focus was placed on the most recent literature to reflect current scientific advancements and novel targeted therapies, foundational older sources were included when they provided essential historical or mechanistic context regarding AD pathogenesis and NK cells.

Additionally, all figures and graphics presented in this manuscript are original concepts designed by the authors and were created using BioRender (BioRender.com).

## 3. NK Cell Pathogenesis and Phenotypic Shifts in AD

### 3.1. Peripheral Blood Alterations and Cutaneous Homing of NK Cells

**Figure 1:**
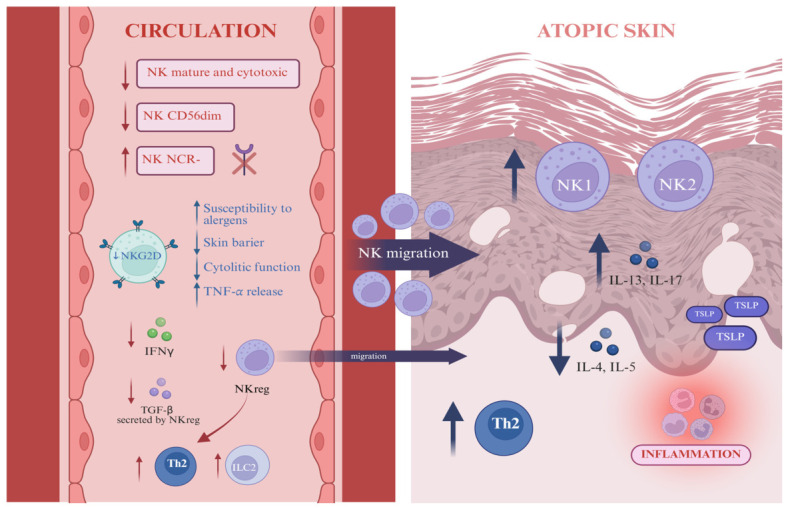
Dynamics of NK cell migration, phenotypic shifts, and inflammatory crosstalk in atopic dermatitis. The provided schematic delineates the phenotypic and functional alterations of immune cells between the circulation and atopic skin. Within the circulation, there is a prominent decrease in mature cytotoxic NK cells, CD56^dim^ subsets, and regulatory NK (NKreg) cells, concomitant with an increase in NCR-NK cells, Th2 response, and ILC2s. Furthermore, the downregulation of the NKG2D receptor is associated with an increased susceptibility to allergens, alongside impaired skin barrier integrity, reduced cytolytic function, and increased TNF-α release. The circulatory compartment also exhibits reduced levels of IFNγ and NKreg-secreted TGF-β. There is a migration of NK cells, especially NK1 and NK2, to the atopic skin. This localized microenvironment is distinguished by the presence of TSLP, elevated concentrations of IL-13 and IL-17, and diminished levels of IL-4 and IL-5, which, alongside an expanded Th2 response ultimately orchestrate a state of localized inflammation [12,13,14,15,16,17,18,19,22]. Abbreviations: IFN-γ—Interferon gamma; IL-4/5/13/17—Interleukin 4/5/13/17; ILC2s—Type 2 Innate Lymphoid Cells; NCR-—Natural Cytotoxicity Receptor-deficient; NK cells—Natural Killer cells; NKreg—Regulatory Natural Killer cells; TGF-β—Transforming Growth Factor beta; Th2—T helper type 2; TNF-α—Tumor Necrosis Factor alpha; TSLP—Thymic Stromal Lymphopoietin; ↑—increase; ↓—decrease. Created in BioRender. Rak, K. (2026) https://BioRender.com/mr8mk4k.

Evidence linking NK cell deficiencies and functional impairments to type 2-driven inflammation suggests that these cells are closely related to the pathogenesis of AD [17]. In the peripheral blood, patients with AD exhibit reduced total NK cell numbers, specifically characterized by a selective loss of mature CD56^dim^ cells and a distinct transcriptional program indicative of activation-induced cell death (AICD) [12]. Studies show a diminished transcriptomic signature of cytotoxic genes in AD, including *GZMA*, *KLRC1*, *IL18RAP*, and *IFNG*. Notably, the most pronounced downregulation occurs in *KLRF1*, which encodes the maturity marker and activating receptor NKp80. Such a selective reduction in NKp80^+^ subpopulations—while markers like CD56, CD16A (FCGR3A), CD94 (KLRD1) and NKG7 remain stable—suggests a specific impairment of mature, cytotoxic NK cells rather than a global depletion of all subsets [13]. This systemic decline is accompanied by the expansion of a nonclassical, natural cytotoxicity receptor-deficient (NCR−) population [12]. Such circulatory dysregulation is particularly evident in pediatric cohorts. Longitudinal analysis of children with AD reveals a progressive accumulation of circulating NK cells with low expression of the activating receptor NKG2D, a profile linked to increased disease severity and sensitivity to both food and aeroallergens. This reduction in NKG2D coincides with persistent allergen sensitization and impaired skin barrier function, as measured by transepidermal water loss [14]. Paradoxically, these low-NKG2D NK cells exhibit depressed cytolytic function alongside an exaggerated release of the pro-inflammatory cytokine TNF-*α*, further fueling the inflammatory cycle [14]. Conversely, in the skin, there is an elevated NK cell transcriptional signature and an increased frequency of NK cells in lesional tissue, likely supported by migration from the blood as an endogenous regulatory response to aberrant type 2 inflammation [12]. According to the studies, the skin of AD patients exhibits a dynamic transcriptional signature characterized by a progressive upregulation of immune signaling pathways, most notably type 2, Th17, and NK cell-related responses [18]. While the type 2 phenotype in lesional skin is primarily driven by IL-13, with IL-4 and IL-5 being virtually absent [18,19], the activation of IL-17 and NK cell signaling serves as a robust transcriptomic feature that discriminates between lesional and non-lesional tissue. This elevated NK cell signature is derived from both NK1 and NK2 subsets, reflecting a complex recruitment of these cells to the cutaneous landscape [18]. This process is particularly pronounced in severe cases of AD. Specifically, in patients with severe AD, the immunological profile is characterized by a significant expansion of both CLA^+^CD56^bright^ and CLA^+^CD56^dim^ NK cell populations. This systemic shift is coupled with an augmented functional capacity. Specifically, CLA^+^CD56^dim^ NK cells demonstrate a heightened expression of IFN-γ, IL-10, and TNF upon stimulation with staphylococcal enterotoxin B (SEB). These findings reinforce the critical role of *S. aureus* enterotoxins in driving the pathogenesis of severe AD. Furthermore, the clinical severity of the disease correlates with an increased dermal expression of NK cell markers, such as NCAM-1/CD56 and pan-granzyme, corroborating the theory of accelerated skin-homing and local cytotoxic activation in the most affected patients [20]. Recent data further indicate that this cutaneous accumulation of NK cells is quantitatively persistent even under systemic treatment, as the global NK-cell-related expression signature remains upregulated despite clinical improvement. While systemic therapy appears to revert the imbalance between resting and activated NK cells, inhibitory receptors on these cells remain persistently elevated. Ultimately, successful therapy is associated with a specific shift in the NK-cell lineage: an increase in CD56 accompanied by a decrease in CD16A and cytotoxic granzymes. This indicates a transition from cytotoxic CD56^dim^CD16^+^ NK cells toward an immunomodulatory CD56^bright^CD16^−^ phenotype as the skin heals [21]. The down-regulation of IFN-γ production and impaired cytotoxicity of NK cells under the influence of the Th2 cytokine microenvironment in atopic dermatitis are schematically illustrated in Figure 1.

Taken together, these findings indicate that AD is not characterized by a simple quantitative deficiency of NK cells. Rather, the disease is associated with a profound redistribution and functional reprogramming of the NK-cell compartment. While mature cytotoxic NK-cell subsets are depleted in the circulation, NK cells accumulate within lesional skin, where they acquire a distinct activation profile shaped by the local inflammatory microenvironment. This phenotypic shift is accompanied by impaired cytotoxicity, altered cytokine production, and persistent changes in NK-cell signaling pathways, suggesting that NK-cell dysfunction in AD reflects a complex process of immune adaptation rather than a mere loss of innate immune effectors.

### 3.2. Regulatory Axes and Immunosuppressive Pathways of NK Cells in AD

**Figure 2:**
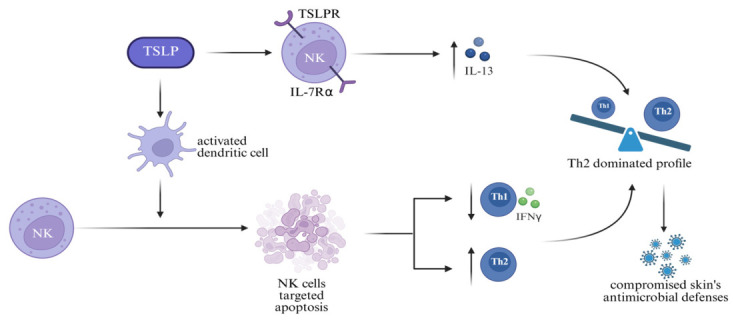
The role of TSLP in AD. TSLP serves a bipartite role in the mechanisms driving the immunological shift between Th1 and Th2 responses. It possesses the capacity to activate dendritic cells, which, upon spatial proximity to NK cells, can predispose these cellular lineages to apoptosis. This cellular depletion markedly diminishes the secretion of Th1-associated cytokines, notably the critical IFN-γ, whilst concurrently amplifying the prominence of the Th2-mediated response. Furthermore, TSLP can directly engage TSLPR and IL-7Rα receptors expressed on the surface of NK cells, thereby upregulating IL-13 secretion and further consolidating a Th2 predominance. Ultimately, these immunological imbalances culminate in the impairment of the skin’s antimicrobial defense mechanisms [15,17,23]. Abbreviations: AD—Atopic Dermatitis; IFN-γ—Interferon gamma; IL-13—Interleukin 13; IL-7Rα—Interleukin-7 Receptor alpha; NK cells—Natural Killer cells; Th1/Th2—T helper type 1/type 2; TSLP—Thymic Stromal Lymphopoietin; TSLPR—Thymic Stromal Lymphopoietin Receptor; ↑—increase; ↓—decrease. Created in BioRender. Rak, K. (2026) https://BioRender.com/g281ves.

The functional capacity of these cells is largely contingent upon their stimulation by activated dendritic cells and the presence of pro-inflammatory TSLP, both of which act as essential signals for NK cell recruitment and subsequent effector function within the cutaneous landscape [17]. However, in the specialized microenvironment of AD-affected tissue, the intimate proximity between NK cells and activated dendritic cells may predispose NK cells to apoptosis. This targeted depletion of the NK cell population not only impairs the production of Th1-type cytokines, but also shifts the immunological balance toward an exaggerated type 2 helper T cell (Th2) response, ultimately compromising the skin’s antimicrobial defenses [23]. When it comes to TSLP, it can either activate dendritic cells to drive Th2-polarized immunity or act directly on NK cells expressing the TSLPR and IL-7Rα subunits. This direct interaction triggers the aberrant production of IL-13, reinforcing the notion that TSLP is a key factor in the functional shift in NK cells throughout the progression of AD [17] [Figure 2]. This immunological deviation is further compounded by a profound deficiency in IFN-γ production, which exacerbates the Th1/Th2 imbalance in favor of a Th2-dominated profile [15]. Beyond the loss of IFN-γ, recent research has identified a novel regulatory NK subset (NKreg) characterized by the production of transforming growth factor beta (TGF-β), which is also significantly depleted in the peripheral blood of AD patients. Identified by the markers CD1d^hi^PD-L1^hi^CD27^+^ in murine models, these cells function as a critical “brake” on allergic inflammation. While they migrate from the circulation to lesional skin, their systemic reduction impairs their ability to suppress the activation of both ILC2s and Th2 effector cells. Adaptive transfer of these TGF-β-producing NK cells has been shown to restore immune homeostasis and improve the regulatory T cell (Treg) to Th2 ratio in lymphoid tissues, suggesting that the loss of this specific regulatory subset is a primary driver of type 2 expansion [22]. This deficiency has significant downstream effects on other innate cells, specifically ILC2s. The interaction between NK cells and ILC2s represents a critical regulatory axis. Research has demonstrated that NK cell activation, predominantly mediated by IFN-γ, inhibits ILC2 amplification and cytokine production both in vitro and in vivo [24]. Consequently, a deficiency in NK cells unleashes these pathogenic ILC2 responses, suggesting that the NK cell–ILC2 inhibition axis is a vital regulatory mechanism for maintaining the skin barrier [12]. Dupilumab, a monoclonal antibody targeting IL-4Rα, mitigates type 2 inflammation and improves skin barrier function by normalizing the aberrant crosstalk between NK cells and ILC2 populations [16]. Recent studies comparing AD patients with and without dupilumab therapy have further illuminated this connection, identifying a high association between circulating NK cells and ILC2s in untreated, moderate-to-severe AD [16]. The suppression of this regulatory axis is increasingly attributed to inhibitory immune pathways. NECTIN2 (CD112/PVRL2), a nectin family adhesion molecule, functions as a ligand for TIGIT and CD112R (PVRIG). This interaction typically suppresses immune activity by reducing IFN-γ production and NK cell cytotoxicity, thereby inhibiting T cell and NK cell proliferation [25]. Research utilizing single-cell transcriptomics reveals that SGK1^hi^ macrophages in AD and psoriasis express NECTIN2, which binds to TIGIT on NK cells and T cells. These findings suggest that this NECTIN2-TIGIT axis acts as a primary driver of immunosuppression, directly stifling NK cell proliferation and inhibiting the production of IFN-γ [26]. Since IFN-γ is a critical suppressor of type 2 inflammation, its absence underscores that NK cell dysregulation is not merely a marker of the disease, but a functionally relevant driver of AD pathology [12]. Consequently, the loss of this potent antiviral cytokine significantly heightens host susceptibility to viral pathogens [15]. NK cells normally suppress Th2/ILC2-driven inflammation, whereas AD is characterized by multiple mechanisms that progressively disable this regulatory function. The mechanistic link between NK cells and the Th17/IL-17 axis is heavily dependent on their crosstalk with dendritic cells. Depending on the microenvironment, NK–dendritic cell interactions can exert a regulatory effect by suppressing autopathogenic Th17 responses through an innate IFN-γ–IL-27 axis [27]. Conversely, NK cells can also activate dendritic cells to promote pathogenic Th17 generation, directly contributing to epithelial barrier disruption [28]. In severe AD, this balance is further disrupted by the progressive accumulation of hyperinflammatory, NKG2D-low NK cells. Although these modified NK cells show reduced cytotoxicity, they exhibit an exaggerated release of pro-inflammatory cytokines, which can amplify dendritic cell-mediated Th17 expansion and exacerbate skin barrier dysfunction [14].

### 3.3. NK Cell Dysregulation as the Initiator of a Vicious Cycle

The profound redistribution and functional reprogramming of NK cells detailed in this section (summarized in Table 1) do not merely skew the immune system toward a Th2-dominated profile; they serve as the catalyst for broader immunological failures. The systemic depletion of mature cytotoxic CD56^dim^ subsets and the critical reduction in IFN-γ production create a localized innate immune deficiency. As will be explored in Section 4, this specific loss of antiviral capacity establishes a permissive environment for viral pathogens. Furthermore, this functionally impaired NK-cell landscape facilitates the unabated colonization of *S. aureus*. In turn, as detailed in Section 5, *S. aureus* actively exploits and exacerbates this very same mature NK-cell depletion, setting the stage for a continuous, vicious cycle of impaired antimicrobial defense and chronic inflammation.

## 4. Predisposition to Infections in Patients with AD

### 4.1. Most Common Infections in Patients with AD

Although epidermal barrier disruption represents a hallmark of atopic dermatitis (AD), the increased susceptibility of patients to viral infections cannot be explained solely by structural skin defects. Accumulating evidence suggests that impaired NK-cell-mediated immunity constitutes a critical component of defective antiviral host defense in AD. As key innate immune effectors, NK cells provide rapid protection against viral pathogens through direct cytotoxic activity and the production of antiviral cytokines, particularly interferon-gamma (IFN-γ). Consequently, the depletion of mature cytotoxic NK-cell subsets, reduced IFN-γ production, and impaired NK-cell activation observed in AD create a permissive environment for viral replication and dissemination [15,29].

Among all infectious complications associated with AD, eczema herpeticum (EH) represents the most compelling clinical manifestation of impaired antiviral immunity. EH affects approximately 3% of patients with AD and is predominantly caused by herpes simplex virus type 1 (HSV-1) [29,30]. The disease is characterized by widespread monomorphic vesiculopustular eruptions, frequently accompanied by fever, lymphadenopathy, and systemic symptoms [30,31,32]. In severe cases, viral dissemination may result in keratoconjunctivitis, hepatitis, encephalitis, or other life-threatening complications [29,30,31]. Several risk factors for EH, including severe AD, early disease onset, elevated serum IgE levels, eosinophilia, and concomitant allergic diseases, overlap with features associated with profound immune dysregulation [29,31,33]. Importantly, NK cells play a pivotal role in the early control of HSV infection, suggesting that the impaired NK-cell responses characteristic of AD may substantially contribute to EH susceptibility. In this context, EH may be viewed as the most clinically apparent consequence of NK-cell dysfunction in AD.

Additional evidence supporting defective antiviral immunity in AD derives from other virus-associated dermatoses. Eczema vaccinatum (EV), a severe complication resulting from exposure to live vaccinia virus, has historically highlighted the inability of some patients with AD to mount effective antiviral immune responses [29,34]. In severe cases, uncontrolled viral dissemination may lead to multiorgan involvement and significant mortality if left untreated [34]. Similarly, eczema coxsackium, caused by enteroviruses of the Coxsackie group, occurs preferentially in individuals with AD and may clinically resemble EH [29,35]. Although the precise contribution of NK cells to these conditions remains incompletely understood, their occurrence further supports the concept of impaired innate antiviral surveillance in AD.

The relationship between AD and viral infections extends beyond acute disseminated viral syndromes. Molluscum contagiosum virus frequently affects patients with AD, partly due to epidermal barrier damage and mechanical autoinoculation resulting from chronic scratching [36,37]. However, the increased prevalence and persistence of molluscum contagiosum in AD also suggest defects in local antiviral immunity. Likewise, human papillomavirus (HPV) infections appear to occur more frequently in individuals with AD, potentially reflecting the combined effects of barrier dysfunction, altered cellular immunity, and impaired interferon-dependent antiviral responses [38,39,40,41].

Importantly, viral susceptibility in AD appears to be closely intertwined with microbial dysbiosis. Clinical studies have identified prior *S. aureus* infection as a risk factor for the development of eczema herpeticum [29,30]. This observation suggests that microbial dysbiosis and antiviral immune dysfunction are not independent phenomena but rather interconnected processes. Given the emerging role of NK cells in both antimicrobial defense and immune regulation, NK-cell dysfunction may represent a mechanistic link connecting *S. aureus* colonization, chronic type 2 inflammation, and impaired antiviral immunity.

Collectively, these findings indicate that susceptibility to viral infections in AD extends beyond a simple consequence of epidermal barrier impairment. Increasing evidence supports a model in which quantitative and functional abnormalities of NK cells compromise early antiviral responses, facilitate viral dissemination, and contribute to the characteristic infectious phenotype of AD. Thus, impaired antiviral immunity represents one of the most clinically relevant manifestations of NK-cell dysfunction and further supports the concept that NK cells constitute a critical link between type 2 inflammation, microbial dysbiosis, and disease progression.

### 4.2. The Intersection of Viral Susceptibility and the Broader AD Cycle

The increased susceptibility to viral infections in AD is intrinsically linked to the phenotypic shifts described in Section 3—most notably, the targeted loss of mature cytotoxic NK-cell subsets and the profound reduction in antiviral IFN-γ. Importantly, this immunodeficiency does not exist in isolation. Clinical observations demonstrating that prior *S. aureus* colonization acts as a significant risk factor for eczema herpeticum highlight a critical intersection between viral susceptibility and bacterial dysbiosis. The defective early antiviral responses discussed here are compounded by *S. aureus*, which, as explored next in Section 5, actively destroys the exact mature NK cells required for viral surveillance. Thus, the infectious phenotype of AD is driven by a vicious cycle: Th2-driven phenotypic alterations combined with bacterial-mediated NK-cell destruction collectively obliterate the host’s antiviral defenses.

## 5. The Skin Microbiome-NK Cell Axis

### 5.1. Physiological Skin Microbiota

The skin microbiota constitutes an integral component of cutaneous immunity, functioning alongside the epidermal barrier and host immune system to prevent microbial invasion. Under physiological conditions, a diverse community of commensal microorganisms occupies distinct ecological niches on the skin surface and contributes to immune homeostasis through competitive exclusion of pathogens, modulation of inflammatory responses, and maintenance of barrier integrity [42,43,44]. Disruption of this balanced ecosystem has been increasingly recognized as an important contributor to the pathogenesis of inflammatory skin diseases, including atopic dermatitis [44,45].

### 5.2. Skin Microbiota in AD

The cutaneous landscape of atopic dermatitis (AD) is fundamentally characterized by a collapse in microbial diversity [45]. In these compromised epidermal zones, *S. aureus* emerges as the dominant resident, with the sheer density of its colonization closely mirroring the clinical severity of the disease [46,47]. Conversely, the surrounding commensal flora, namely coagulase-negative *Staphylococcus* spp., *Streptococcus* spp., and *Corynebacterium*, frequently exhibit targeted antimicrobial activity to suppress *S. aureus* proliferation [45]. *Cutibacterium*, however, demonstrates a paradoxical duality within this ecosystem. While capable of inhibiting *S. aureus* growth through glycerol fermentation, specific strains actively facilitate the pathogen’s colonization, aggregation, and subsequent biofilm construction [48,49]. Adding further complexity to these microbial dynamics, Gonzalez et al. established that the overarching population volumes of *Cutibacterium*, *Streptococcus*, and *Corynebacterium* remain notably consistent across healthy control, lesional, and nonlesional cohorts. Nevertheless, a focused analysis of the worst lesional microenvironments reveals a stark, localized depletion in both *Cutibacterium* and *Corynebacterium* colonization [46].

Beyond bacterial shifts, the cutaneous mycobiome frequently exhibits enhanced fungal colonization in individuals afflicted with AD [44]. *Malassezia* species dominate this fungal landscape, typically residing as benign commensals under normal physiological conditions [50]. However, species-level profiling reveals distinct compositional divergences between healthy and diseased skin. Specifically, lesional areas demonstrate a marked proliferation of *M. sloofiae* and *M. dermatis*, whereas the relative abundance of *M. globosa* and *M. restricta* remains remarkably stable across both patient and control cohorts [51]. The clinical relevance of *Malassezia* in the pathogenesis of this dermatosis is profound; its presence is inextricably linked to an amplified immune response, as evidenced by the concurrent elevation of both total and *Malassezia*-specific immunoglobulin E (IgE) levels [45,52].

Importantly, these alterations are unlikely to represent a purely passive consequence of epidermal barrier disruption. Increasing evidence suggests that microbial dysbiosis and innate immune dysfunction are closely interconnected processes, with NK cells emerging as potential regulators of this relationship.

### 5.3. Direct Action of S. aureus on NK Cells

**Figure 3:**
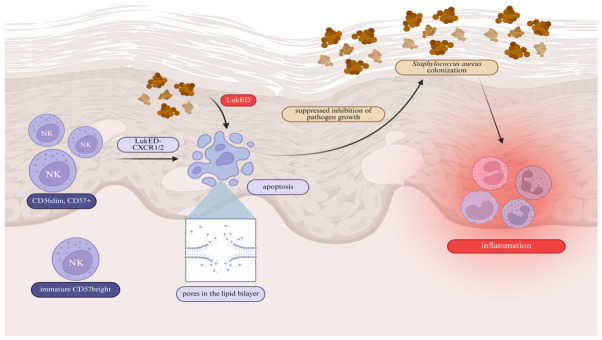
The role of *Staphylococcus aureus* in AD. *S. aureus* significantly contributes to the pathogenesis of atopic dermatitis (AD) through the elaboration of the LukED toxin. By engaging the CXCR1/2 cascade, this toxin initiates pore formation within the lipid bilayer of Natural Killer (NK) cells, resulting in the selective apoptotic lysis of mature CD56dim and CD57+ subsets. Notably, the CD56bright population is specifically spared by the pathogen and escapes this apoptotic fate. The subsequent depletion of mature NK cells compromises cutaneous antimicrobial defense mechanisms, thereby facilitating progressive *S. aureus* colonization and perpetuating chronic skin inflammation [46,53,54,55,56,57,58]. Abbreviations: AD—Atopic dermatitis; CD56bright/CD56dim/CD57+—Differentiation markers of Natural Killer cell subsets; CXCR1/CXCR2—Chemokine receptors utilized by the LukED toxin; LukED—Leukocidin ED; NK—Natural Killer cells; *S. aureus*—*Staphylococcus aureus*. Created in BioRender. Rak, K. (2026) https://BioRender.com/qr7dg6k.

The pathogenic success of *S. aureus* is largely predicated on its sophisticated secretory arsenal, replete with cytotoxic and immunomodulatory agents designed to subvert host immunity. A ubiquitous hallmark of this evasive strategy is the elaboration of pore-forming leukocidins, toxins that specifically target and compromise the lipid bilayers of host immune cells [53]. Recent work by Boulouis et al. illuminates the profound vulnerability of mucosa-associated invariant T (MAIT) cells, a diverse population of unconventional, non-MHC-restricted T lymphocytes, to leukocidin ED (LukED) induced lysis [54,55]. These cells undergo devastatingly rapid destruction at negligible toxin concentrations via a CCR5-dependent cascade, a pathway distinctly separate from the CXCR1- and CXCR2-driven mechanism LukED utilizes to neutralize NK cells [55,56,57,58]. The targeted eradication of NK cells via the LukED-CXCR1/2 cascade critically undermines the local microenvironment’s capacity to suppress pathogenic overgrowth. Consequently, this precise immune subversion facilitates entrenched *S. aureus* colonization, serving as a vital catalyst that propels the host tissue toward unrelenting chronic inflammation in AD [58]. Intriguingly, *S. aureus* employs a highly discriminatory cytolytic strategy: the toxin selectively eradicates mature, highly cytotoxic NK cell subsets, specifically the CD56^dim^ and CD57+ populations. The deliberate sparing of the immature CD56^bright^ NK cell pool suggests a calculated evolutionary tactic, wherein the pathogen prioritizes dismantling immediate, fully functional immunological threats. These dual pathways of LukED-mediated cell lysis, highlighting the CXCR1/2-dependent destruction of mature NK cells alongside the CCR5-targeted elimination of MAIT cells, are schematically represented in Figure 3. This receptor-level adaptability underscores the sophisticated, multi-pronged nature of the *S. aureus* offensive arsenal [55]. Viewed through this lens, the microbiome dysbiosis seen in AD, particularly the pathological expansion of *S. aureus* [46], emerges not as a mere passive byproduct of barrier disruption, but as an active, orchestrated subversion of the host’s localized immune defenses [55].

Beyond superficial colonization, the capacity of *S. aureus* to invade keratinocytes and generate robust extracellular biofilms establishes a highly effective physical barrier against immune surveillance [59,60]. Furthermore, analyses of AD skin biopsies have revealed a phenotypic shift of the pathogen into small colony variants (SCVs), which are characterized by increased antibiotic resistance and diminished immunogenicity. By concurrently upregulating host autophagy and facilitating inflammasome degradation, *S. aureus* significantly attenuates its recognition by the immune system, thereby securing intracellular survival and establishing persistent colonization [60,61]. Consequently, this multifaceted evasion strategy renders the bacteria spatially inaccessible to the cytotoxic mechanisms of NK cells, effectively neutralizing the local innate immune responses required for microbial clearance [59,60].

### 5.4. Pro-Inflammatory NK Cells: A New Research Area

Importantly, NK-cell dysfunction in AD should not be viewed exclusively as a loss of cytotoxic activity. Emerging evidence suggests that under specific inflammatory conditions, NK cells may undergo functional reprogramming toward aberrant pro-inflammatory phenotypes.

In a recent, seminal publication, Davies et al. revealed that systemic *S. aureus* infection drives the robust proliferation of an aberrant CD57-NKG2A+ NK cell subset [62]. Phenotypically, the marker profile of this subset indicates an immature developmental state. CD57 expression is a well-established indicator of late-stage maturation in NK cells, correlating with high cytotoxic potential due to elevated intracellular levels of perforin and granzyme B [63]. In contrast, the absence of the CD57 marker, coupled with the retention of the inhibitory NKG2A receptor, characterizes a cell population that lacks robust cytotoxic capacity. Instead, these immature CD57- cells exhibit a significantly higher proliferative potential when exposed to inflammatory cytokines [63].

While their investigation primarily focused on the bloodstream, these findings offer a compelling conceptual framework for exploring analogous mechanisms within the cutaneous microenvironment of AD, a condition inherently burdened by intense *S. aureus* colonization [46,62]. Specifically, the CD57-NKG2A+ NK cells identified in bacteremic patients exhibited an augmented propensity to release mediators that actively fuel inflammatory pathogenesis, a phenotypic shift orchestrated by staphylococcal superantigens [62].

The precise mechanism driving the robust expansion of this specific subset relies on indirect, cytokine-mediated signaling rather than direct superantigen-to-NK-cell ligation. Staphylococcal superantigens, such as SEA, SEB, and TSST-1, initiate systemic immune activation by cross-linking major histocompatibility complex (MHC) class II molecules on antigen-presenting cells with the variable regions of T-cell receptors. This dual interaction triggers massive polyclonal T-cell proliferation and an overwhelming release of inflammatory mediators [64,65]. The resulting cytokine storm selectively expands the immature CD57- NK cell pool. Because these less differentiated NK cells exhibit a substantially higher baseline transcription of cytokine receptors—such as the IL-12 receptor—they are intrinsically more responsive to the cytokine surge than their terminally differentiated CD57+ counterparts, granting them a distinct proliferative advantage [63].

Because *S. aureus* is a ubiquitous colonizer of the epidermis, physical breaches in the skin can inadvertently act as conduits for the pathogen to enter systemic circulation and precipitate bacteremia [66]. In the context of AD, this risk is profoundly amplified; a thoroughly compromised epidermal barrier exacerbated by excoriation from scratching, structural filaggrin defects, broad immunological dysregulation, and a fundamental dearth of antimicrobial peptides readily facilitates such bacterial translocation [67,68]. Synthesizing these observations reveals a provocative mechanistic parallel: the identical staphylococcal superantigens (including TSST-1, SEA, and SEB) implicated in the systemic expansion of NK CD57-NKG2A+ cells are prolifically secreted by *S. aureus* within the cutaneous milieu. This striking convergence provides a compelling rationale for future investigations to determine whether a localized, superantigen-driven skewing of the NK cell compartment occurs directly within the skin [62,67].

Crucially, the emergence of this pro-inflammatory subset does not contradict the immunodeficiencies described in Section 3.2; rather, they coexist and synergistically drive disease chronicity. As the chronic AD microenvironment and *S. aureus* toxins selectively drive mature, highly cytotoxic subsets and regulatory NKreg cells into apoptosis, the immunological mechanisms that typically suppress allergic inflammation are abolished [22]. Concurrently, the superantigen-induced cytokine storm selectively expands the remaining pool of immature CD57-NKG2A+ NK cells via their highly expressed cytokine receptors. This superantigen-driven replacement of regulatory and cytotoxic NK cells with a highly proliferative, hyperinflammatory subset directly amplifies dendritic cell-mediated Th17 expansion, locking the host in a vicious cycle of persistent inflammation and barrier dysfunction.

### 5.5. Closing the Vicious Cycle of S. aureus and NK Cell Subversion

The active subversion of NK cells by *S. aureus* serves as the final, critical link in the self-amplifying loop of AD pathogenesis. By selectively targeting mature CD56dim subsets for LukED-mediated lysis and driving the aberrant expansion of pro-inflammatory phenotypes via superantigens, *S. aureus* directly enforces the very phenotypic shifts and functional impairments identified in Section 3. Consequently, by actively eradicating the specific mature NK cells necessary for both bacterial clearance and viral surveillance, *S. aureus* not only secures its own persistent colonization, but also firmly establishes the permissive environment for viral dissemination discussed in Section 4. Ultimately, this intricate interplay—where type 2 inflammation alters NK cell function, paving the way for *S. aureus* which then actively dismantles remaining mature NK cells to further cripple antiviral defenses—constitutes a continuous vicious cycle that drives disease chronicity.

### 5.6. Not Only the Skin Microbiome

Recent clinical explorations into gut microbiota transplantation (FMT) and its refined derivative, washed microbiota transplantation (WMT), offer profound insights into the functional crosstalk between intestinal homeostasis and cutaneous immunity in atopic dermatitis (AD). Emerging evidence indicates that the characteristic epidermal dominance of *S. aureus* may be intimately tied to underlying dysregulations within the Natural Killer (NK) cell compartment, thereby highlighting the formidable, albeit intricate, influence of the gut-skin axis in shaping host defenses [69,70].

Strategically restoring intestinal eubiosis precipitates a marked expansion of microbial taxa proficient in synthesizing short-chain fatty acids (SCFAs), most notably, *Lachnospiraceae*, *Coprococcus*, and *Eubacterium coprostanoligenes* [69]. This gastrointestinal metabolic shift propagates a highly coordinated systemic immune cascade that directly revitalizes NK cell populations. Specifically, as elucidated by Liu et al., the engraftment of a healthy microbiome fundamentally upregulates the expression of perforin and IL-12p70 on NK cells. This molecular shift signals the robust recovery of their innate cytolytic proficiency, occurring in tandem with a systemic downregulation of the aberrant Th2 and Th17 inflammatory axes [70,71].

Critically, this gut-driven restoration of NK cell cytotoxicity mirrors a profound remodeling of the pathological epidermal niche. As systemic immunity stabilizes, the cutaneous microenvironment transitions away from an *S. aureus*-dominated state, fostering the rebuilding of protective commensals such as *Acinetobacter* and *Perlucidibaca* [69,70]. Collectively, these immunological and microbial shifts illuminate the gut–skin axis not merely as a secondary observational phenomenon, but as a premier frontier for future investigation, harboring immense potential for new therapeutic strategies in the clinical management of AD.

Taken together, current evidence suggests that microbial dysbiosis and NK-cell dysfunction are not independent features of AD but rather mutually reinforcing processes. *S. aureus* directly impairs NK-cell survival and function, while defective NK-cell-mediated antimicrobial surveillance promotes persistent microbial colonization. This self-amplifying cycle may represent a critical mechanism linking type 2 inflammation, microbial dysbiosis, and disease chronicity in AD.

## 6. Restoring NK-Cell Homeostasis in Atopic Dermatitis: Current Evidence and Future Perspectives

The growing recognition of NK-cell dysfunction as a contributor to type 2 inflammation, microbial dysbiosis, and impaired antiviral immunity has generated increasing interest in therapeutic strategies capable of restoring NK-cell homeostasis in atopic dermatitis (AD). Unlike traditional approaches that primarily target downstream inflammatory pathways, interventions aimed at correcting NK-cell abnormalities may simultaneously influence multiple pathogenic domains of the disease, including immune regulation, antimicrobial defense, and viral susceptibility.

### 6.1. Direct Restoration of NK-Cell Function: IL-15-Based Approaches

Among emerging therapeutic strategies, augmentation of IL-15 signaling represents the most direct approach to NK-cell restoration. IL-15 is a critical cytokine regulating NK-cell development, survival, proliferation, and cytotoxic activity. In a landmark study, Mack et al. demonstrated that administration of an IL-15 superagonist (IL-15 SA) in a murine model of AD induced a marked expansion of circulating and cutaneous NK cells. This increase was accompanied by a substantial reduction in pathogenic ILC2 and eosinophil populations, resulting in significant improvement of both clinical and histopathological disease parameters. Importantly, the therapeutic effect was entirely dependent on NK cells, as it persisted in Cd8−/− mice but was abolished following conditional NK-cell deletion. These findings provide compelling evidence that restoration of NK-cell activity can directly suppress type 2 inflammation and ameliorate AD-like disease [12].

### 6.2. Indirect Restoration of NK-Cell Homeostasis Through Type 2 Cytokine Blockade

Although currently approved biologic therapies do not directly target NK cells, growing evidence indicates that successful suppression of type 2 inflammation may secondarily restore NK-cell homeostasis. Dupilumab, a monoclonal antibody targeting IL-4Rα, represents the best-studied example of this phenomenon. Beyond its well-established clinical efficacy, dupilumab has been shown to normalize aberrant interactions between NK cells and ILC populations, particularly ILC2s, which are central drivers of type 2 inflammation. Treatment attenuates excessive NK–ILC crosstalk, likely reflecting improved barrier function and reduced antigen-driven immune activation within lesional skin [16].

Importantly, dupilumab also appears to reverse systemic NK-cell abnormalities. Patients with moderate-to-severe AD exhibit depletion of mature CD56dim NK cells alongside expansion of dysfunctional NCR− populations. Following treatment, the pathological NCR− subset is markedly reduced, while both total NK-cell numbers and mature CD56dim populations recover toward levels observed in healthy individuals [12]. These observations suggest that NK-cell dysfunction in AD is not irreversible but rather represents a dynamic consequence of the inflammatory microenvironment.

### 6.3. Microbiome-Directed Therapies and the Gut–Skin–NK Axis

The emerging concept of the gut–skin axis has provided additional insight into the therapeutic modulation of NK-cell function. Studies investigating fecal microbiota transplantation (FMT) and washed microbiota transplantation (WMT) demonstrate that restoration of intestinal eubiosis promotes the expansion of short-chain fatty acid-producing bacterial taxa and induces systemic immunological changes associated with enhanced NK-cell activity. Increased expression of perforin and IL-12-related pathways following microbiota restoration suggests improved NK-cell cytotoxic capacity, accompanied by suppression of Th2 and Th17 inflammatory responses [69,70,71].

These systemic effects appear to extend to the skin, where microbial restoration is associated with reduced *S. aureus* dominance and partial recovery of commensal microbial communities. Such findings support the hypothesis that microbiome-targeted interventions may indirectly restore NK-cell-mediated antimicrobial surveillance and contribute to disease control.

### 6.4. Future Directions

Several promising therapeutic avenues remain largely unexplored. Targeting inhibitory immune checkpoint pathways, including the NECTIN2–TIGIT axis, may represent a strategy for reversing NK-cell suppression and restoring IFN-γ production. Similarly, approaches aimed at preserving or expanding regulatory NK-cell subsets (NKreg) could potentially re-establish physiological control over ILC2 and Th2 responses. Advances in microbiome engineering, precision immunotherapy, and NK-cell-directed biologics may further expand the therapeutic landscape in the coming years.

## 7. Challenges and Limitations

Despite growing evidence supporting a role for NK-cell dysfunction in the pathogenesis of atopic dermatitis (AD), several important challenges continue to limit the translation of these findings into clinical practice.

One of the major obstacles is the substantial gap between experimental models and human disease. Although IL-15-based approaches have demonstrated remarkable efficacy in murine models of AD, important biological differences exist between murine and human NK-cell compartments, including their phenotypic diversity, tissue distribution, and migratory behavior. Consequently, the therapeutic potential of NK-cell-directed interventions observed in animal studies may not be directly reproducible in patients.

A further limitation relates to the difficulty of studying tissue-resident NK cells in human skin. Most currently available data are derived from peripheral blood analyses, whereas the local cutaneous microenvironment is likely to be the primary site where NK cells influence disease activity. The low abundance of tissue-resident NK cells, technical challenges associated with skin biopsy processing, and the lack of standardized isolation and characterization protocols continue to hinder a comprehensive understanding of NK-cell biology within lesional skin.

Another unresolved issue concerns the causal relationship between NK-cell dysfunction and AD pathogenesis. Current evidence consistently demonstrates associations between disease activity and alterations in NK-cell number, phenotype, and function. However, it remains unclear whether these abnormalities represent a primary pathogenic driver or develop secondarily as a consequence of chronic type 2 inflammation, barrier disruption, and microbial dysbiosis. The observation that therapies such as dupilumab can restore NK-cell homeostasis supports the reversibility of these changes, yet does not definitively establish causality.

Additional uncertainty surrounds the therapeutic manipulation of NK cells. While restoration of NK-cell function may improve antimicrobial and antiviral immunity, excessive activation of the NK-cell compartment could potentially promote unwanted inflammatory responses. Strategies based on IL-15 signaling, immune checkpoint modulation, or NK-cell expansion therefore require careful evaluation with regard to safety, dosing, and long-term immunological consequences.

Finally, several mechanistic questions remain unresolved. The precise role of tissue-resident NK cells, the contribution of regulatory NK-cell subsets (NKreg), the functional significance of the NECTIN2–TIGIT axis, and the interplay between NK cells, *S. aureus* colonization, and microbial dysbiosis all warrant further investigation. Similarly, the therapeutic relevance of microbiome-directed interventions and their capacity to restore NK-cell homeostasis remain largely unexplored in prospective clinical studies.

Addressing these limitations will require integrated translational research combining advanced single-cell technologies, spatial transcriptomics, longitudinal patient cohorts, and interventional clinical studies. Such efforts will be essential to determine whether NK-cell dysfunction represents a biomarker of disease activity or a therapeutically actionable driver of AD pathogenesis.

## 8. Conclusions

The traditional view of atopic dermatitis (AD) as a disease driven predominantly by adaptive type 2 immunity is increasingly being challenged by emerging insights into the role of innate immune dysregulation. The evidence summarized in this review (comprehensively outlined in Table 2) supports the concept that NK-cell dysfunction represents a central yet underappreciated component of AD pathogenesis, linking three major hallmarks of the disease: type 2 inflammation, microbial dysbiosis, and impaired antiviral immunity.

Rather than reflecting a simple numerical deficiency, NK-cell abnormalities in AD encompass a complex process of phenotypic redistribution, functional reprogramming, and impaired immunoregulatory capacity. The depletion of mature cytotoxic NK-cell subsets, reduced IFN-γ production, disruption of the NK–ILC2 regulatory axis, and loss of antimicrobial surveillance collectively contribute to the maintenance of chronic inflammation and increased susceptibility to infections. At the same time, microbial factors, particularly *S. aureus*, actively exacerbate NK-cell dysfunction, establishing a self-amplifying cycle that promotes disease persistence.

Importantly, growing evidence suggests that these abnormalities are not irreversible. The restoration of NK-cell homeostasis observed following targeted blockade of type 2 inflammation, together with emerging data from microbiome-directed interventions and experimental IL-15-based therapies, highlights the therapeutic potential of modulating the NK-cell compartment. Beyond improving skin inflammation, such approaches may offer the additional benefit of strengthening antimicrobial and antiviral host defenses.

Taken together, current findings position NK cells as a key mechanistic bridge between epidermal barrier dysfunction, microbial dysbiosis, and immune dysregulation in atopic dermatitis (AD). Targeted translational and clinical efforts are necessary to determine whether NK cell dysfunction represents merely a biomarker of disease activity or a true driver. Future studies should consider routine NK cell phenotyping as a potential predictive biomarker of patient response in trials of new biologics. In parallel, clinical trials investigating the efficacy of direct NK cell modulation, for example, using IL-15 agonists, are warranted. Such a focused and integrated approach will ultimately validate their pathogenic significance and facilitate the development of a new generation of innovative therapies targeting innate immunity in the treatment of AD.

## Figures and Tables

**Table 1:** Major quantitative and functional abnormalities of NK cells in atopic dermatitis and their pathogenic consequences.

NK-Cell Alteration	Proposed Mechanism	Functional Consequence	Clinical Relevance	References
Reduced CD56^dim^ NK cells	AICD, chronic activation	impaired cytotoxicity	increased infection susceptibility	[12]
Reduced NKp80+ cells	loss of mature NK subsets	impaired effector responses	severe disease phenotype	[13]
Reduced NKG2D expression	chronic inflammatory signaling	decreased antiviral activity	allergen sensitization, barrier dysfunction	[14]
Expansion of NCR−NK cells	phenotypic reprogramming	dysfunctional NK responses	chronic inflammation	[12]
Reduced IFN-γ production	TSLP, TIGIT pathways	loss of Th2 suppression	enhanced type 2 inflammation	[12,15,25,26]
Reduced NKreg cells	impaired TGF-β signaling	increased ILC2 and Th2 activity	disease exacerbation	[22]
Increased skin homing of NK cells	CLA-mediated migration	local immune activation	lesional inflammation	[12,18,20,21]

Abbreviations: NK—Natural Killer, AICD—activation-induced cell death, NCR-—Natural Cytotoxicity Receptor-deficient, IFN-γ—interferon gamma, TSLP—thymic stromal lymphopoietin, NKreg—Regulatory Natural Killer cells, TGF-β—transforming growth factor beta, ILC2—type 2 innate lymphoid cells; TIGIT—T-cell immunoreceptor with Ig and ITIM domains; CLA—cutaneous lymphocyte-associated antigen.

**Table 2:** NK-cell dysfunction as the missing link between type 2 inflammation, microbial dysbiosis and antiviral susceptibility in AD.

Pathogenesis Phase	Key Factors/Cells	Phenotypic Changes & Mechanisms	Consequences	References
Initiation (Type 2 inflammation)	Barrier defects, Th2, ILC2, dendritic cells, TSLP	↑ IL-4, IL-13, IL-31. Activation of TSLPR and IL-7Rα on NK cells (IL-13 secretion)	Chronic inflammation, barrier disruption, NK cell apoptosis	[6,7,15,17,23]
NK-cell dysfunction	NK cells, SGK1hi macrophages	Blood: ↓ CD56dim, ↓ NKp80+, ↓ NKG2D, ↑ TNF-α, ↑ NCR−, ↓ IFN-γ, ↓ NKreg (↓ TGF-β). Skin: Influx of CLA+CD56bright and CLA+CD56dim, ↑ NECTIN2-TIGIT axis	Failure to suppress ILC2 and Th2. Profound immunosuppression and decreased NK cell proliferation	[12,13,14,15,18,20,21,22,24,25,26]
Clinical consequences (Dysbiosis and infections)	Microbiome (*S. aureus*, Cutibacterium, Corynebacterium, Malassezia), viruses	Decreased microbiome diversity. Dominance of *S. aureus*, ↓ Cutibacterium and Corynebacterium, ↑ M. sloofiae and M. dermatis	Susceptibility to complications: EH, EV, Eczema coxsackium, molluscum contagiosum virus, HPV	[29,30,31,32,33,34,35,36,37,38,39,40,41,44,45,46,47,48,49,50,51,52]
Vicious cycle	*S. aureus* (LukED, SCVs, superantigens)	Lysis of CD56dim and CD57+ via CXCR1/CXCR2. Phenotypic shift to SCVs. Expansion of pro-inflammatory NK cells (CD57-NKG2+) stimulated by superantigens	Active destruction of local defenses. Persistent colonization perpetuating inflammation	[46,53,54,55,56,57,58,59,60,61,62,67]
Therapeutic interventions	IL-15 SA, Dupilumab, FMT, WMT	Targeted therapies: NK cell expansion, ↑ CD56dim, ↓ NCR−, normalization of the NK-ILC2 axis. Microbiome: ↑ SCFAs upregulate perforin and IL-12p70 expression on NK cells	Restoration of NK-cell homeostasis and cytotoxicity. Suppression of Th2/Th17 responses, resolution of *S. aureus* dominance	[12,16,69,70,71]

Abbreviations: AD—Atopic dermatitis; CD1dhiPD-L1hiCD27+—Surface markers identifying NKreg cells; CD56bright/CD56dim/CD57+—Differentiation markers of Natural Killer cell subsets; CLA—Cutaneous lymphocyte-associated antigen; CXCR1/CXCR2—Chemokine receptors utilized by the LukED toxin; EH—Eczema herpeticum; EV—Eczema vaccinatum; FMT—Fecal microbiota transplantation; HPV—Human papillomavirus; HSV-1—Herpes simplex virus type 1; IFN-γ—Interferon-gamma; IL—Interleukin (e.g., IL-4, IL-13, IL-15, IL-31); IL-12p70—Interleukin-12 subunit p70; IL-15 SA—IL-15 superagonist; ILC2—Type 2 innate lymphoid cells; LukED—Leukocidin ED; NCR−—Natural cytotoxicity receptor-deficient; NECTIN2—Nectin family adhesion molecule (CD112/PVRL2); NK—Natural Killer cells; NKG2D—Activating receptor of NK cells; NKp80+—Maturity marker and activating receptor of NK cells (encoded by KLRF1); NKreg—Regulatory NK cells; SCFA—Short-chain fatty acid; SCV—Small colony variant; SEA/SEB—Staphylococcal enterotoxin A/B; TGF-β—Transforming growth factor beta; Th2—Type 2 helper T cells; TIGIT—T cell immunoreceptor binding the NECTIN2 molecule; TNF-α—Tumor necrosis factor-alpha; TSLP—Thymic stromal lymphopoietin; TSST-1—Toxic shock syndrome toxin-1; WMT—Washed microbiota transplantation; ↑—increase; ↓—decrease.

## Data Availability

No new data were created or analyzed in this study. Data sharing is not applicable.
